# Molecular Details of Olfactomedin Domains Provide Pathway to Structure-Function Studies

**DOI:** 10.1371/journal.pone.0130888

**Published:** 2015-06-29

**Authors:** Shannon E. Hill, Rebecca K. Donegan, Elaine Nguyen, Tanay M. Desai, Raquel L. Lieberman

**Affiliations:** School of Chemistry & Biochemistry, Georgia Institute of Technology, Atlanta, Georgia, United States of America; University of Iowa, UNITED STATES

## Abstract

Olfactomedin (OLF) domains are found within extracellular, multidomain proteins in numerous tissues of multicellular organisms. Even though these proteins have been implicated in human disorders ranging from cancers to attention deficit disorder to glaucoma, little is known about their structure(s) and function(s). Here we biophysically, biochemically, and structurally characterize OLF domains from *H*. *sapiens* olfactomedin-1 (npoh-OLF, also called noelin, pancortin, OLFM1, and hOlfA), and *M*. *musculus* gliomedin (glio-OLF, also called collomin, collmin, and CRG-L2), and compare them with available structures of myocilin (myoc-OLF) recently reported by us and *R*. *norvegicus* glio-OLF and *M*. *musculus* latrophilin-3 (lat3-OLF) by others. Although the five-bladed β-propeller architecture remains unchanged, numerous physicochemical characteristics differ among these OLF domains. First, npoh-OLF and glio-OLF exhibit prominent, yet distinct, positive surface charges and copurify with polynucleotides. Second, whereas npoh-OLF and myoc-OLF exhibit thermal stabilities typical of human proteins near 55°C, and most myoc-OLF variants are destabilized and highly prone to aggregation, glio-OLF is nearly 20°C more stable and significantly more resistant to chemical denaturation. Phylogenetically, glio-OLF is most similar to primitive OLFs, and structurally, glio-OLF is missing distinguishing features seen in OLFs such as the disulfide bond formed by N- and C- terminal cysteines, the sequestered Ca^2+^ ion within the propeller central hydrophilic cavity, and a key loop-stabilizing cation-π interaction on the top face of npoh-OLF and myoc-OLF. While deciphering the explicit biological functions, ligands, and binding partners for OLF domains will likely continue to be a challenging long-term experimental pursuit, we used structural insights gained here to generate a new antibody selective for myoc-OLF over npoh-OLF and glio-OLF as a first step in overcoming the impasse in detailed functional characterization of these biomedically important protein domains.

## Introduction

Olfactomedins comprise a large protein family (PFAM: PF02191) with seven phylogenetic branches [1]. These multidomain proteins contain a ~30 kDa olfactomedin (OLF) domain and are predominantly expressed extracellularly in a variety of tissues of multicellular organisms, particularly in vertebrates as well as selected invertebrates [2]. Although the specific biological functions, binding partners, and mechanism(s) of action still remain largely unknown, the involvement of OLF domain-containing proteins in diseases is broadly documented, particularly in the case of glaucoma [3], but also in a host of cancers [2], inflammatory bowel disorder and Crohn’s/colitis [4], defense against infection [5], attention deficit disorder [6], and childhood obesity [7].

The difficulty in assigning discrete biological function to OLFs is due in part to the fact that in general, reports of partial deletion mutants or knock-out mice of a variety of OLF domain-containing proteins do indicate a strong phenotype, e.g. gross abnormalities or systemic disease [8–11]. This observation, combined with considerable sequence similarity [1], has suggested that OLF domains might exhibit somewhat compensatory functions [2]. However, the extents of such overlap, or interconnectedness in function and/or binding partners, remain major open questions. For example, we recently reported the crystal structure of the best studied OLF domain from myocilin (myoc-OLF) [12], a protein linked to inherited forms of glaucoma in populations throughout the world. The leading proposed pathogenic mechanism involves intracellular aggregation leading to cell death in the trabecular meshwork, a tissue of the eye implicated in maintaining pressure; high pressure is a major risk factor for glaucoma [3]. Myoc-OLF variants are exquisitely prone to misfolding, corresponding aggregates exhibit characteristics of amyloid *in vitro* [13, 14] and in cells [14], and have aberrant interactions with molecular chaperones [15, 16]. However, the lack of myocilin does not cause glaucoma, and in spite of considerable research efforts over the past 20 years, the normal functional role of wild type myocilin in the trabecular meshwork remains unclear [3].

The availability of the *H*. *sapiens* myoc-OLF 5-bladed β-propeller structure, along with two others recently reported, (*R*. *norvegicus* gliomedin (glio-OLF) [17] and *M*. *musculus* latrophilin-3 (lat3-OLF) [18]), should provide new insight and enable the development of selective reagents, such as antibodies or small molecules, to better probe OLF function and pathophysiology. Here we present biophysical, biochemical, and structural characterization of the OLF domain of a second glio-OLF, that from *M*. *musculus* (94% identical to *R*. *norvegicus*), and *H*. *sapiens* noelin/pancortin/olfm1/hOlfA (npoh-OLF), which are both neuronal OLFs, but from different phylogenetic subfamilies [1]. Gliomedin, also called collomin, collmin, and CRG-L2, is involved in the development of the peripheral nervous system and is phylogenetically most similar to invertebrate OLFs [1]. The gliomedin extracellular region, which contains two collagen domains and one OLF domain, is shed from its membrane tether [19]. The emerging functional picture is that this truncated species trimerizes via the collagen domains, binds to the first three fibronectin-III-like domains of neurofascin 186 to recruit axonal sodium channels and thus facilitates the formation of, and helps maintain, the Nodes of Ranvier [10, 19–21]. Olfactomedin-1 has been linked to neurogenesis and neural crest formation [22, 23], cortex development [24], valve formation in the developing embryo heart [25], spheroid attachment onto endometrial cells [26], and formation of actin stress fibers in podocytes [27]. Olfactomedin-1 has been shown to interact with select binding partners [8, 28], of which just two involve binding to the olfactomedin-1 OLF domain [24, 29]. We identify key physicochemical differences among recently solved OLF structures that we then exploit to create an antibody selective for myoc-OLF over glio-OLF and npoh-OLF. Our work expands our appreciation of the divergent biophysical properties that likely confer largely unique protein interactions among OLF domains from different subfamilies and lays further groundwork for developing reagents to understand the extent of redundant biological functions of this enigmatic yet important domain family.

## Materials and Methods

### Expression, purification, and biophysical characterization

The OLF domains of olfm1 (Genebank accession No: BC011741.2, residues 218–485) and gliomedin (Genebank accession No: NP_796324.1, residues 279–549) were amplified (5-PRIME Master Mix, Fisher Scientific) from *H*. *sapiens* olfactomedin-1 DNA (Open Biosystems, clone ID 3352603) and *M*. *musculus* gliomedin DNA (Open Biosystems, clone ID 40058551), respectively. The amplified products were annealed into pET-30 Xa/LIC (Novagen) and subcloned into the pMAL-c4x vector (New England Biolabs) along with the Factor Xa recognition sequence between maltose binding protein (MBP) and the OLF domain, as previously described for myoc-OLF [30]. To improve protein expression, the npoh-OLF(C221G) variant was generated by site directed mutagenesis (QuikChange, Stratagene). All plasmids were verified by DNA sequencing (MWG Operon). Primers are listed in S1 Table.

Npoh-OLF and glio-OLF plasmids were transformed into *E*. *coli* Rosetta-gami 2(DE3)pLysS (Novagen) cells. Procedures used for cell growth and protein expression were similar to methods used to prepare myoc-OLF [30] except the Superior Broth (U.S. Biological) was supplemented with 1 mM CaCl_2_ for npoh-OLF and 0.1 mM isopropyl β-D-1-thiogalactopyranoside concentration was used to induce protein expression. Cell lysis and purification protocols were also similar to those described for myoc-OLF [30], with the following modifications: the lysis buffer contained 0.1 mg/mL bovine pancreas deoxyribonuclease I (DNAse, Sigma) and 5 mM CaCl_2_/MgCl_2_, and the buffers for chromatographic steps (amylose affinity, gel filtration) after Factor Xa cleavage were composed of 50 mM *N*-cyclohexyl-2-aminoethanesulfonic acid (CHES) pH 8.6, 150 mM NaCl without or with 10 mM maltose for amylose wash/gel filtration and amylose elution, respectively. Overall yield was ~0.1 mg purified npoh-OLF(C221G) and ~0.02 mg purified glio-OLF per L cell culture.

### Crystallization, data collection, structure determination

Both npoh-OLF(C221G) (8 mg/ml in 50 mM CHES pH 8.6, 150 mM NaCl) and glio-OLF (3.6 mg/mL in 50 mM CHES pH 8.6, 150 mM NaCl) grew crystals by the sitting drop method at 16°C. For npoh-OLF, the reservoir solution was composed of 100 mM Hepes pH 7.5, 25% PEG 400, 3% PEG 3000 and 10% glycerol, and for glio-OLF the solution contained 100 mM phosphate citrate pH 5.5 and 25% PEG 600. Npoh-OLF crystals were cryo-cooled in mother liquor directly while for glio-OLF, 4 μl of a 50% glycerol solution was added to the crystallization drop (initial volume 2 μl, equal volume protein and reservoir) immediately prior to crystal harvesting. Data were collected at the Southeast Regional Collaborative Access Team (SER-CAT) 22-ID beamline and processed using HKL-3000 [31]. The npoh-OLF structure was solved by molecular replacement using Phaser [32] with a homology model generated from myoc-OLF in SwissPDBviewer [33] and the glio-OLF structure with a npoh-OLF model generated by Phenix Sculptor [34]. The models were iteratively built and refined using Coot [35] and Phenix.refine [32]. Data collection and refinement statistics are presented in Table 1; structures have been deposited to the protein databank with PDB codes 4XAT and 4XAV.

**Table 1:** Crystallographic Data Collection and Refinement Statistics (values in parentheses are for the highest resolution shell).

	Npoh-OLF	Glio-OLF
**Data collection**		
Space group	P 2_1_2_1_2_1_	P 2_1_
Cell dimensions *a*, *b*, *c* (Å)	39.68, 68.02, 95.66	47.16, 140.64, 78.15
*α*,*β*,*γ* (°)	90, 90, 90	90, 106.03, 90
Resolution (Å)	32.27–2.11	44.65–2.05
*R*_merge_	0.0865 (0.2553)	0.1214 (0.4200)
*I*/σ*I*	9.4 (2.7)	9.1 (2.7)
Completeness (%)	97.9 (89.5)	98.2 (95.0)
Redundancy	3.1 (2.5)	5.2 (4.9)
**Refinement**		
Resolution (Å)	32.27–2.11	44.65–2.05
No. of unique reflections	46741 (3453)	59953 (5781)
*R*_work/_ *R*_free_	0.1560/0.2088	0.1662/0.2137
No. atoms		
* *Protein	2069	7900
* *Ligand/ion	14	62
* *Water	137	802
Average B-factors (Å^2^)		
* *Protein	24.9	23.2
* *Ligand/ion	42.6	33.4
* *Water	32.0	31.2
R.m.s deviations		
* *Bond lengths (Å)	0.013	0.005
* *Bond angles (°)	1.39	0.92
Ramachandran favored and additional allowed	100	99.8
MolProbity [60] score (percentile)	1.94 (85^th^)	1.62 (95^th^)

### Structure analysis

Structural alignments were conducted using SSM [36] using the website PDBeFold (http://www.ebi.ac.uk/msd-srv/ssm). Sequence alignments were prepared using PROMALS3D [37] and rendered in ESPript [38], which uses DSSP [39] for secondary structure assignment. Electrostatic surfaces were calculated using APBS [40] and figures generated in PyMOL (www.pymol.org) using default secondary structure assignment.

### Chemical unfolding

Intrinsic tryptophan fluorescence of cleaved npoh-OLF or glio-OLF (0.7 μM) in 10 mM sodium phosphate dibasic/potassium phosphate monobasic, 200 mM NaCl pH 7.2 was measured using a Shimadzu RF-530/PC spectrofluorophotometer under varying denaturing concentrations of guanidinium hydrochloride (GdHCl, 0 to 5 M) or urea (0 to 8 M). After overnight incubation at room temperature for each sample condition, four spectra were averaged and buffer subtracted using an excitation wavelength of 284 nm (slit width, 5 nm) and an emission range 300–500 nm (slit width, 5 nm). Each data point is an average of three independent measurements, excluding glio-OLF in urea, which was only performed once due to the lack of an unfolding transition.

### Metal ion identification

The effects of Ca^2+^ on thermal stability of npoh-OLF (C221G) and glio-OLF were measured as described for myoc-OLF [41] using differential scanning fluorimetry. Briefly, cleaved proteins were prepared in 50 mM CHES pH 8.6, 150 mM NaCl in the presence or absence of 10 mM CaCl_2_, MgCl_2_, Ca(OAc)_2_, Mg(OAc)_2_, or KCl and the midpoints of unfolding, i.e. melting temperatures (T_m_s), were calculated [41]. For the detection of calcium, the fluorescence of the nanomolar affinity Ca^2+^ chelator Quin-2 was measured under native and denaturing conditions as previously described for myoc-OLF [41]. Samples containing 8 μM npoh-OLF(C221G) or glio-OLF, 150 μM Quin-2, and either 0 or 1.4 M GdHCl (npoh-OLF(C221G)), and 0 or 5 M GdHCl (glio-OLF) in 50 mM CHES pH 8.6, 150 mM NaCl buffer were measured using a Biotek Synergy 2 plate reader (excitation, 360/40 nm; emission, 528/20 nm). Reported fluorescence values are an average of two samples, blank subtracted, and recorded after 45 minutes of incubation at room temperature.

### Heparin binding, nucleotide extraction, and lipid binding

Using a HiTrap Heparin HP column (GE Healthcare), myoc-OLF and npoh-OLF(C221G) heparin binding was measured in 20 mM CHES pH 8.6 with 20 mM NaCl or 10 mM KH_2_PO_4_/Na_2_HPO_4_ pH 7.2 with 200 mM NaCl and an elution gradient up to 2 M NaCl in the respective buffer. Heparin binding by myoc-OLF was also tested in 10 mM sodium acetate pH 4.6 with 10 mM CaCl_2_. Thermal stability assays were conducted as described above, in the presence or absence of 0.75 mg/mL heparin sulfate, chondroitin sulfate, or hyaluronic acid in 10 mM Hepes pH 7.5, 200 mM NaCl.

Nucleotides were isolated from monomeric MBP-npoh-OLF(C221G) purified without adding DNAse to the lysis buffer, using established methods for phenol-chloroform extraction and ethanol precipitation [42]. To a solution of purified MBP-npoh-OLF(C221G) in a microcentrifuge tube an equal volume of 25:24:1 phenol:chloroform:isoamyl alcohol (Fisher Scientific) at pH 6.7 was added. The solution was vortexed for one minute and separated via centrifugation with a tabletop microcentrifuge at 15,000 x g for 5 min. The aqueous layer was removed and placed in a new microcentrifuge tube to which 200 μL of EB buffer (Qiagen) was added. The sequence of vortexing, centrifugation, and removal of aqueous layer was then repeated. The aqueous fractions were subsequently washed by 1:1 dilution of 24:1 chloroform:isoamyl alcohol (Amresco) followed by 1 minute of vortexing and 5 minutes of centrifugation. The aqueous layer was removed for ethanol precipitation. 5 M NH_4_OAc (Fisher Scientific) was added to final concentration of 0.75 M, and the solution was mixed. Next, chilled 100% ethanol (Koptec) at a volume of 2.5 x the aqueous layer was added, incubated at -20°C for 1 hr, and centrifuged at 15,000 x g for 20 min. The ethanol was decanted, and any remaining ethanol was allowed to evaporate. The pellet was washed with 50 μL of 80% ethanol, vortexed and re-pelleted at 15,000 x g for 15 min. The washing process was performed twice before the final pellet was allowed to air dry and suspended in EB buffer (Promega). The presence of nucleotides, and possible contamination by phenol and protein, were monitored by measuring absorption at 260 nm, 270 nm, and 280 nm, respectively, with a Biotek Synergy 2 plate reader using a Take3 plate. Nucleotides were visualized via 8% Urea-PAGE and stained with Sybr Green dye (Lonza).

Lipid binding was tested using a membrane lipid array (Echelon Biosciences) with both purified fusion protein MBP-npoh-OLF(C221G) or npoh-OLF(C221G) domain alone devoid of nucleotides, according to the manufacturer’s protocol. The primary antibody for experiments with MBP-npoh-OLF(C221G) was a monoclonal mouse anti-MBP antibody (Santa Cruz, sc13564) and that for npoh-OLF was rabbit polyclonal anti-noelin C-terminal antibody (Abcam, ab151416), both diluted 1:5,000. The anti-noelin antibody recognized our purified npoh-OLF protein (see Results and Discussion), which was spotted on the lipid array membrane as a positive control. Secondary antibodies were horseradish peroxidase-conjugated goat anti-mouse for assays using MBP-npoh-OLF, or goat anti-rabbit for assays using npoh-OLF(C221G) (Kirkegaard & Perry Laboratories), diluted 1:2,000.

### Antibody production and dot blots

Custom rabbit polyclonal antibodies were raised against the *M*. *musculus* myocilin peptide sequence R_332_YELDTETVKAEKEIPGA (Fisher Scientific). Crude serum at a dilution of 1:1,000 was then used to test the specificity of this custom myocilin antibody when compared to commercial antibodies: (1) rabbit polyclonal anti-myocilin H130 raised against *H*. *sapiens* amino acids 240–370 (Santa Cruz Biotechnology, sc-20976) at a 1:500 dilution and (2) rabbit polyclonal anti-noelin C-terminal antibody raised against *H*. *sapiens* noelin amino acids 421–485 (Abcam, ab151416) at a 1:1,000 dilution. For the immunoblot, 2 μL of 9 μM and 2 μL of 3 μM npoh-OLF(C221G), myoc-OLF, and glio-OLF were spotted separately onto a pre-wet PVDF membrane and allowed to air dry. The membranes were blocked in 5% nonfat milk overnight, followed by a washing step and subsequent incubation for 1 hour with primary antibody. The secondary antibody, horseradish peroxidase-conjugated goat anti-rabbit (Kirkegaard & Perry Laboratories), was incubated for 1 hour at 1:500 for anti-myocilin H130 and 1:1,000 for anti-noelin and the custom myocilin antibody. Blots were developed with chemiluminescent horseradish peroxidase detection reagent (Denville).

## Results and Discussion

**Fig 1:**
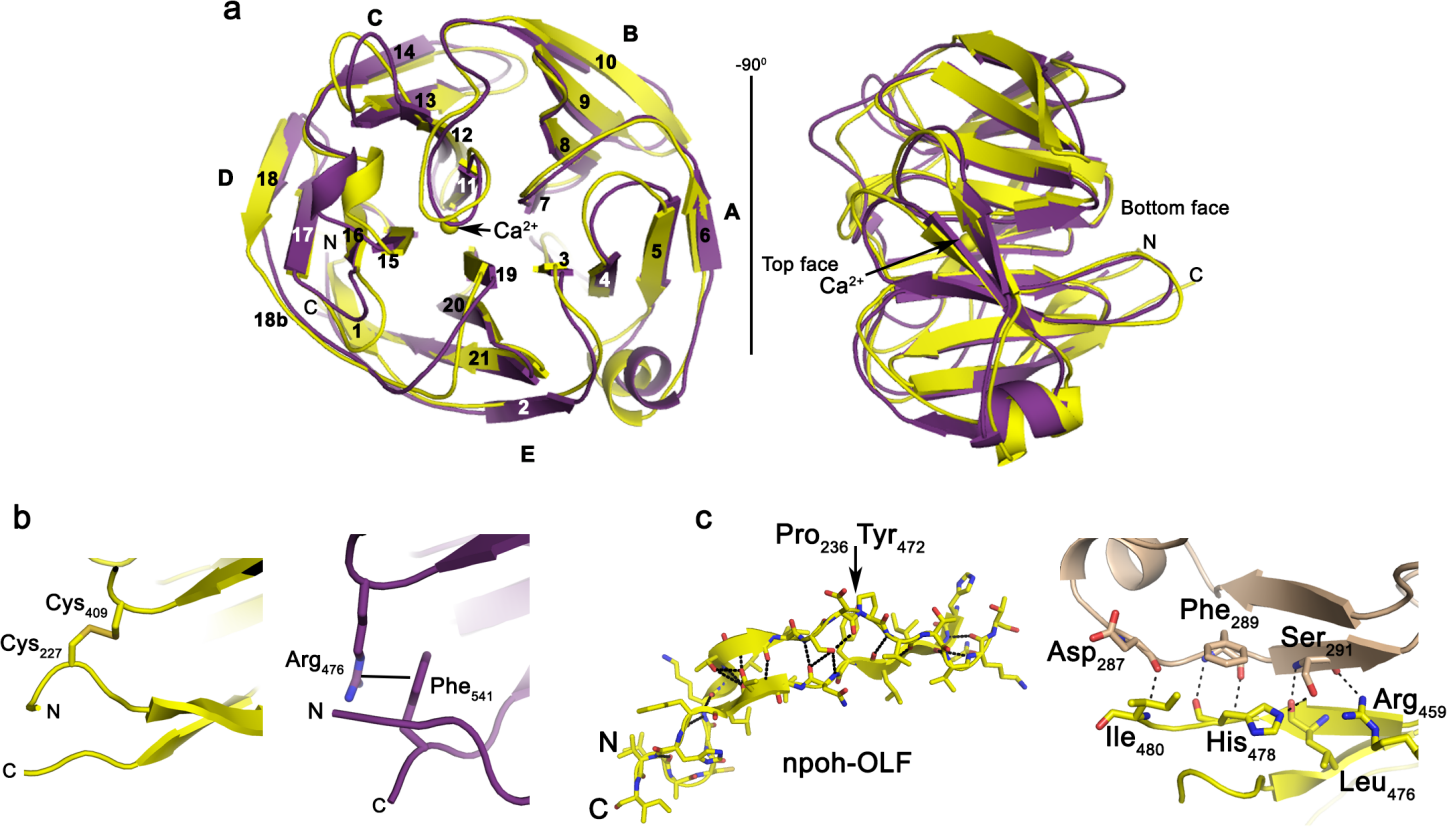
Structural features of npoh-OLF (yellow) and glio-OLF (purple). (a) Overlay of npoh-OLF and glio-OLF in two orientations with strands and blades labeled (r.m.s.d. over Cα atoms is 1.456 Å). (b) Disulfide bond in npoh-OLF (left) and corresponding cation-π interaction in glio-OLF (right). (c) Overview of molecular clasp region highlighting Pro, Tyr residues discussed in text; polar contacts < 3.5 Å are depicted as black dashes (left). Crystal contact of npoh-OLF involving residues from the molecular clasp and an outer strand of blade A from an adjacent symmetry-related molecule (right).

Our *E*. *coli* expression and purification strategy for myoc-OLF involving fusion to MBP via a short cleavable linker was transferrable to both *M*. *musculus* glio-OLF and *H*. *sapiens* npoh-OLF. For npoh-OLF, the point mutation C221G, before the start of the structural OLF domain, increased yields sufficient for crystallization (not shown). The 2.1 Å resolution structure npoh-OLF(C221G), and the 2.05 Å resolution structure of glio-OLF (Materials and Methods, Table 1) were solved by molecular replacement using search models derived from myoc-OLF and npoh-OLF, respectively. The final models include npoh-OLF residues 225–480 (numbering scheme for full-length *H*. *sapiens* olfactomedin-1) and glio-OLF residues 299–542 (numbering scheme for full-length *M*. *musculus* gliomedin). Npoh-OLF crystallized as a monomer in the asymmetric unit in space group P2_1_2_1_2_1_, while glio-OLF crystallized in space group P2_1_ with four molecules in the asymmetric unit consisting of nearly identical chains (root mean squared deviation (r.m.s.d.) of Cα atoms between each pair of chains ≤ 0.35 Å, not shown). Our structure of *M*. *musculus* glio-OLF was solved independently and in a different space group from that of *R*. *norvegicus* OLF (PDB code 4D77) [17]. Any differences in molecular interactions involving non-conserved amino acids among mouse, rat, and human glio-OLF are explicitly stated. Npoh-OLF exhibits the expected OLF five-bladed β-propeller architecture with a short α-helix (Fig 1A); no major conformational changes are seen between our mouse glio-OLF structure and the previously reported rat glio-OLF. Although pairwise sequence similarity is apparent (S1 Fig) and is 40% or greater, comparison of npoh-OLF (phylogenetic subfamily [1] I), glio-OLF (subfamily VI), myoc-OLF (subfamily III), and lat3-OLF (subfamily II, PDB code 5AFB) structures reveals common and dissimilar features, with implications for the OLF domain family at large.

Differences in secondary structure among the solved structures occur primarily in loops at the top and bottom face of the propeller where sequences are most divergent [12] and thus cannot be reliably predicted by homology modeling, as well as in the lengths of β-strands within blades D and E (Fig 1A). Of the two features of OLF that stabilize the propeller in closed circular conformation, namely, a disulfide bond found at the bottom face (Fig 1B and S1 Fig) and a molecular clasp [43], both are preserved in npoh-OLF, but only the latter is present in glio-OLF. Human and mouse glio-OLF lack these corresponding Cys residues, and the disulfide bond is replaced with a cation-π interaction involving Arg 476, at an equivalent position to Cys 409 in npoh-OLF, and Phe 541, located within the C-terminal loop immediately after strand E-21 (Fig 1B and S1 Fig). The cation-π is not strictly conserved in glio-OLF, however, as in select sequences, such as in rat glio-OLF, Arg 476 is replaced with Gln, and in other glio-OLF sequences, Phe 541 is replaced with a Leu or Val (not shown). The molecular clasp involves extensive hydrogen bonding interactions from the sequentially-discontinuous two outer strands of blade E, labeled E-1-E-2 (N-terminus) and E-21 (C-terminus) in npoh-OLF (Fig 1A and 1C). Npoh-OLF has a clasp that extends longer than that of the other OLFs because it forms a crystal contact with the outermost strand of blade A from a neighboring molecule (Fig 1C). An N-terminal proline creates an apparent β-bulge [44] that opposes a C-terminal tyrosine, which contributes polar contacts within a largely hydrophobic interface between blades D and E, and, in myoc-OLF, is the site of a highly destabilizing [45], glaucoma-causing mutation, Y437H [46]. Interestingly, in lat3-OLF, the bulge is not preserved because the proline is not conserved (not shown).

**Fig 2:**
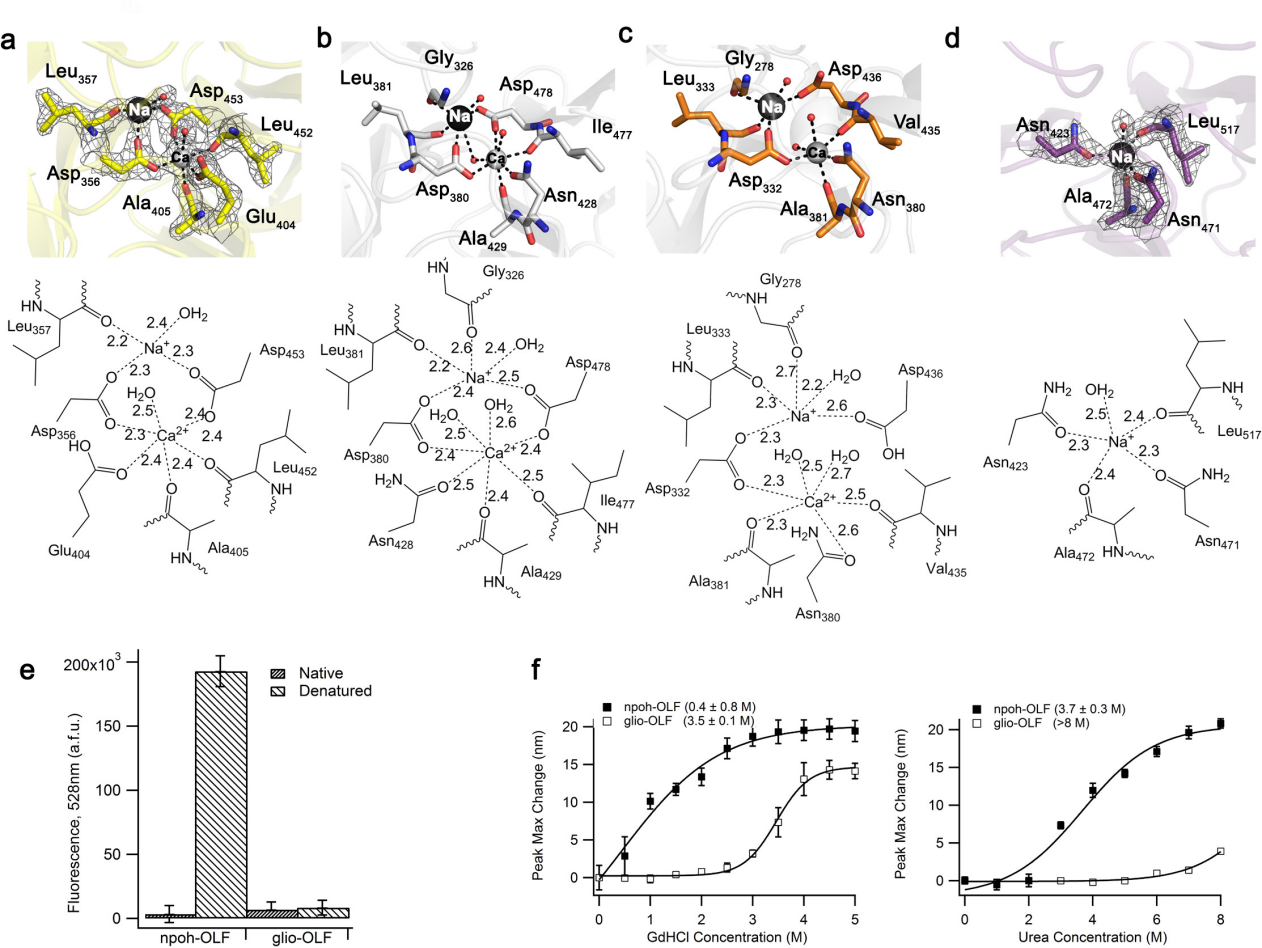
Comparison of metal ion binding sites for four OLFs and biophysical analysis for npoh-OLF and glio-OLF. (a) Metal binding sites in npoh-OLF. (b) Metal binding sites in myoc-OLF (PDB code 4WXU). (c) Metal binding sites in lat3-OLF (PDB code 5AFB). (d) Metal binding site in *M*. *musculus* glio-OLF. Lower panels show interacting distances ≤ 2.7 Å. For (a), (d), 2F_o_-F_c_ electron density is contoured at 1σ. (e) Quin-2 fluorescence (in a.f.u., arbitrary fluorescence units) due to Ca^2+^ binding under native and denaturing conditions. Denaturing concentrations were 1.4 M GdHCl for npoh-OLF, and 5 M GdHCl for glio-OLF. (f) Chemical unfolding curves of npoh-OLF and glio-OLF monitored by the change in maximum intrinsic tryptophan fluorescence using GdHCl (left) and urea (right). Concentration at unfolding midpoint indicated in parentheses.

The OLF domain propeller blades are arranged radially around a central hydrophilic cavity. In npoh-OLF, ~13 Å below the top surface loop covering the entrance to the cavity (Fig 1, and see below), a 6-coordinate ion is found, ligated by the side chains of Asp 356, Glu 404, and Asp 453, as well as the backbone carbonyls of Ala 405 and Leu 452, and one water molecule (Fig 2A). This Ca^2+^ site is superimposable with the previously identified 7-coordinate Ca^2+^ binding motif of myoc-OLF [12] (Fig 2B). The coordinating residues are nearly identical; replacement of Asn 428 in myoc-OLF for Glu 404 in npoh-OLF likely provides charge stabilization that removes the need for a second coordinating water molecule. The site is also somewhat similar to that modeled in lat3-OLF, but to a lesser extent than myoc-OLF because the npoh-OLF Asp 453 equivalent residue in lat3-OLF, Asp 436, is not coordinated to the metal ion, resulting in octahedral geometry but with only four protein-derived ligands (Fig 2C). Unlike the lat3-OLF calcium binding site, whose identity was based on computational considerations [18], the corresponding myoc-OLF site was previously confirmed as Ca^2+^ by metal analysis and characterized as largely inaccessible to chelators such as the high affinity, Ca^2+^-specific fluorescent EGTA analog Quin-2 [41]. Similarly, for npoh-OLF, Ca^2+^, but not Mg^2+^ or K^+^, confers thermal stability (Table 2), and the Ca^2+^ ion is resistant to chelation by Quin-2, except under denaturing conditions (Fig 2E). These experimental results lend strong biochemical credibility to the npoh-OLF metal assignment. Adjacent to the Ca^2+^ site in npoh-OLF, at a distance of ~3.9 Å and similar to the case of myoc-OLF [12] (Fig 2B), a second major *F*_o_-*F*_c_ difference electron density peak was found and modeled as a Na^+^ ion coordinated by Asp 356 and Asp 453, as well as the carbonyl backbone of Leu 357 and a water molecule (Fig 2A).

By contrast, key amino acids present in the expected metal binding region in glio-OLF render it unable to form a coordination sphere for Ca^2+^ (Fig 2D). At the analogous position of npoh-OLF Asp 453 is Ser 518 in mouse and rat glio-OLF (an alanine in the human sequence) (S1 Fig). In both mouse and rat glio-OLF structures, an apparent Na^+^ ion is coordinated by Asn 423, Asn 471, Ala 472, Leu 517, and a water molecule (Fig 2D). In our structure, this ion assignment was made based on lack of stabilization by Ca^2+^, Mg^2+^, or K^+^ in solution (Table 2), unresponsiveness to Quin-2 (Fig 2E), fit of electron density, and refined metal-ligand distances consistent with Na^+^ [47]. Interestingly, in spite of the lack of a Ca^2+^ site, thermal stability of glio-OLF is substantially higher than the other OLFs measured to date (Table 2 and [30, 45]), and glio-OLF is considerably more resistant to chemical unfolding than myoc-OLF [48] and npoh-OLF (Fig 2F and see below). The apparent lack of Ca^2+^ binding for glio-OLF supports our previous conclusion that the primary function of this propeller is likely not Ca^2+^-mediated catalysis [12]. Knowledge of the glio-OLF structure should prompt specific probing of a biological role for the apparent cavity-bound Na^+^, as it may be present to tailor the function of gliomedin in developing the sodium channel-rich nodes of Ranvier in myelinating axons involved in the propagation of electrical signals [10]. For glio-OLF, there is no evidence for any additional metal ions within the hydrophilic cavity.

**Table 2:** Analysis of Thermal Stabilization of OLF domains.

Sample	T_m_ (°C)	ΔT_m_ (°C)
npoh-OLF	58.8 ± 0.1	—-
npoh-OLF + 10mM CaCl_2_	67.0 ± 0.1	8.2
npoh-OLF + 10mM Ca(OAc)_2_	67.4 ± 0.1	8.6
npoh-OLF + 10mM MgCl_2_	54.9 ± 0.1	-3.9
npoh-OLF + 10mM Mg(OAc)_2_	54.0 ± 0.2	-4.8
npoh-OLF + 10mM KCl	58.7 ± 0.2	-0.1
glio-OLF	69.7 ± 0.1	—-
glio-OLF + 10mM CaCl_2_	69.3 ± 0.2	-0.4
glio-OLF + 10mM Ca(OAc)_2_	69.5 ± 0.1	-0.2
glio-OLF + 10mM MgCl_2_	69.3 ± 0.1	-0.4
glio-OLF + 10mM Mg(OAc)_2_	69.6 ± 0.1	-0.1
glio-OLF + 10mM KCl	69.8 ± 0.2	0.1
npoh-OLF^a^	59.6 ± 0.2	—-
npoh-OLF + heparin sulfate^a^	63.3 ± 0.2	3.9
npoh-OLF + chondroitin sulfate^a^	63.8 ± 0.1	4.4
npoh-OLF + hyaluronic acid^a^	64.4 ± 0.1	5.0
glio-OLF^a^	74.7 ± 0.5	—-

^a^T_m_ measured in 10 mM Hepes, 200 mM NaCl pH 7.5 (Buffer A) with or without 0.75 mg/mL of GAG. For myoc-OLF, T_m_ is ~53°C in Buffer A [61].

**Fig 3:**
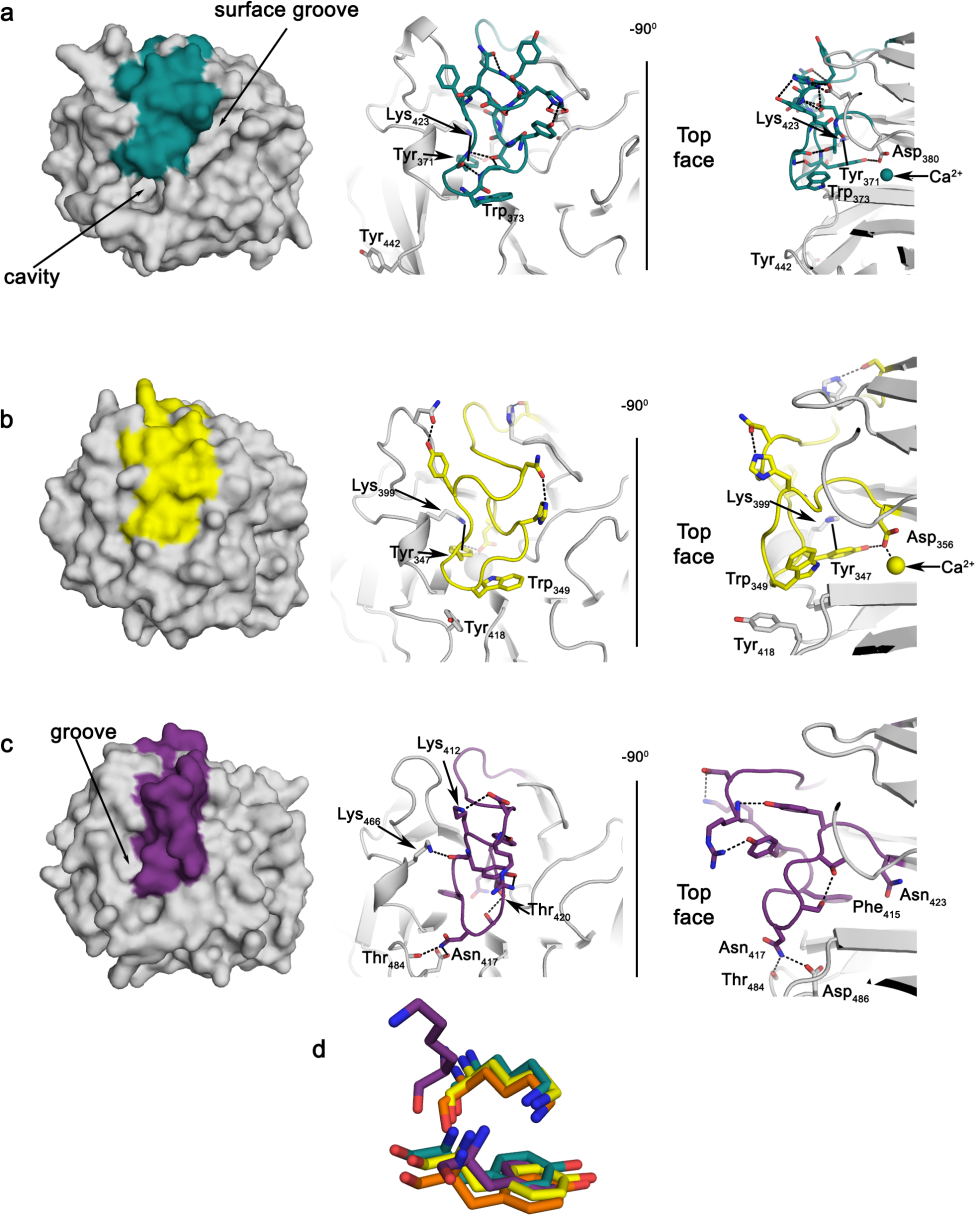
Comparison of top surface features, loop B-10/C-11, and related cation-π interaction. Comparison of (a) myoc-OLF (PDB code 4WXU), (b) npoh-OLF, and (c) *M*. *musculus* glio-OLF. Left: surface representation of loop region; right: key side chain interactions presented as sticks in two orientations. (d) Overlay of Lys/Tyr cation-π interaction conserved for myoc-OLF (blue-green), npoh-OLF (yellow), and lat3-OLF (orange) but disrupted for glio-OLF (purple).

Another characteristic of OLFs is a long, well-defined loop found at the top face, a common site for protein-protein interactions [49], which connects strands B-10 to C-11 and caps the top entrance to the central cavity (Fig 3A, 3B and 3C). This loop is well preserved in structure (Fig 1A), yet only weakly in sequence (S1 Fig) among OLF structures solved to date, but its importance is underscored by the finding that residues stabilizing loop B-10/C-11 are the most conserved among OLFs. In myoc-OLF, numerous point mutations both within and involved in stabilizing loop B-10/C-11 lead to amyloid aggregation and moderate to severe early onset glaucoma [12]. For myoc-OLF, access to the central cavity appears gated by the movements of Trp 373 in this loop and Tyr 442 in an adjacent loop connecting strands D-16/D-17 (Fig 3A) [12]. An analogous pair, Trp 349/Tyr 418, is found within npoh-OLF, and in the structure appears to be in a conformation that seals off the cavity (Fig 3B). By comparison, the lat3-OLF loop containing the equivalent of myoc-OLF Tyr 442 is not visible in electron density and thus not modeled [18], indicating it may be highly mobile and cavity access is not restricted (not shown). In glio-OLF, analogous residues to the Trp/Tyr pair are Asn 417/Asp 486, which are hydrogen bonded; this cross-loop interaction is further stabilized by hydrogen bonding between Asn 417 and the main chain carbonyl oxygen of Thr 484 on loop D-16/D-17 (Fig 3C). Thus, the available glio-OLF structures appear to be in a closed conformation that prevents access to the central cavity (Fig 3C).

A second feature of loop B-10/C-11 common to myoc-OLF, npoh-OLF, and lat3-OLF, but not seen in glio-OLF, is a highly conserved cation-π interaction buried just below the loop formed by Lys 423 and Tyr 371 in myoc-OLF (Fig 3A). Myoc-OLF variants K423E and Y371D are associated with severe cases of glaucoma [50], and K423E is one of the least stable variants [45], supporting the importance of this feature in maintaining the structural integrity of the domain. In npoh-OLF the corresponding residues are Lys 399 and Tyr 347 (Fig 3B) and the interaction is part of an extended hydrogen-bonding network; the hydroxyl group of the Tyr stabilizes the carboxylate side chain of Asp 356, which helps position this residue for coordination to Ca^2+^ (Fig 3B, and see above). Surprisingly, the cation-π interaction is missing in glio-OLF. The corresponding lysine residue, Lys 466, is now solvent exposed in all available glio-OLF structures, stabilizes Asp 410 within loop B-10/C-11 (Fig 3C and 3D). In our structure Lys 466 participates in a phosphate-mediated crystal contact (not shown). The entire loop harboring Lys 466, D-16/D-17, is also shifted away from loop B-10/C-11, creating a small groove (Fig 3C). In glio-OLF, Phe 415 is the residue corresponding to Tyr 371 (myoc-OLF)/Tyr 347 (npoh-OLF), which cannot form a hydrogen bonding interaction with Asn 423, the Asp 380 (myoc-OLF)/Asp 356 (npoh-OLF) equivalent (Fig 3A, 3B, and 3C).

Based on the above characterization, glio-OLF is an outlier in several respects: it lacks the ability to chelate Ca^2+^, does not have a disulfide bond, nor does it have the cation-π interaction described for loop B-10/C-11. Yet, glio-OLF is highly resistant to denaturation thermally and chemically (Table 2 and Fig 2F). This finding is surprising when compared to myoc-OLF, where essentially all documented non-synonymous variants of myoc-OLF that do not occur in surface-exposed loops are glaucoma-causing [51], destabilized [30], and prone to aggregation [12–14, 52]. Comparison of hydrophobic interactions [53] among glio-OLF, npoh-OLF, and myoc-OLF reveals at least 20 more hydrophobic interactions for glio-OLF. Some of the additional hydrophobic interactions in glio-OLF include interactions between blades A and E (Leu 311 and Leu 353), as well as a number of interactions within strand B-10 and loop B-10/C11 (Leu 401, Leu 407, and Tyr 421). This finding is consistent with prior engineering efforts for β-propellers that have demonstrated the importance of hydrophobic interactions for structural stability of the fold [54]. An intriguing question for future study, then, is why do animals have high levels of aggregation–prone myoc-OLF in the context of full-length myocilin in the eye, an organ that experiences UV exposure and other environmental conditions known to be detrimental to protein stability, when there exists a more primitive [1] glio-OLF, with features that could, in principle, better resist such stressors.

**Fig 4:**
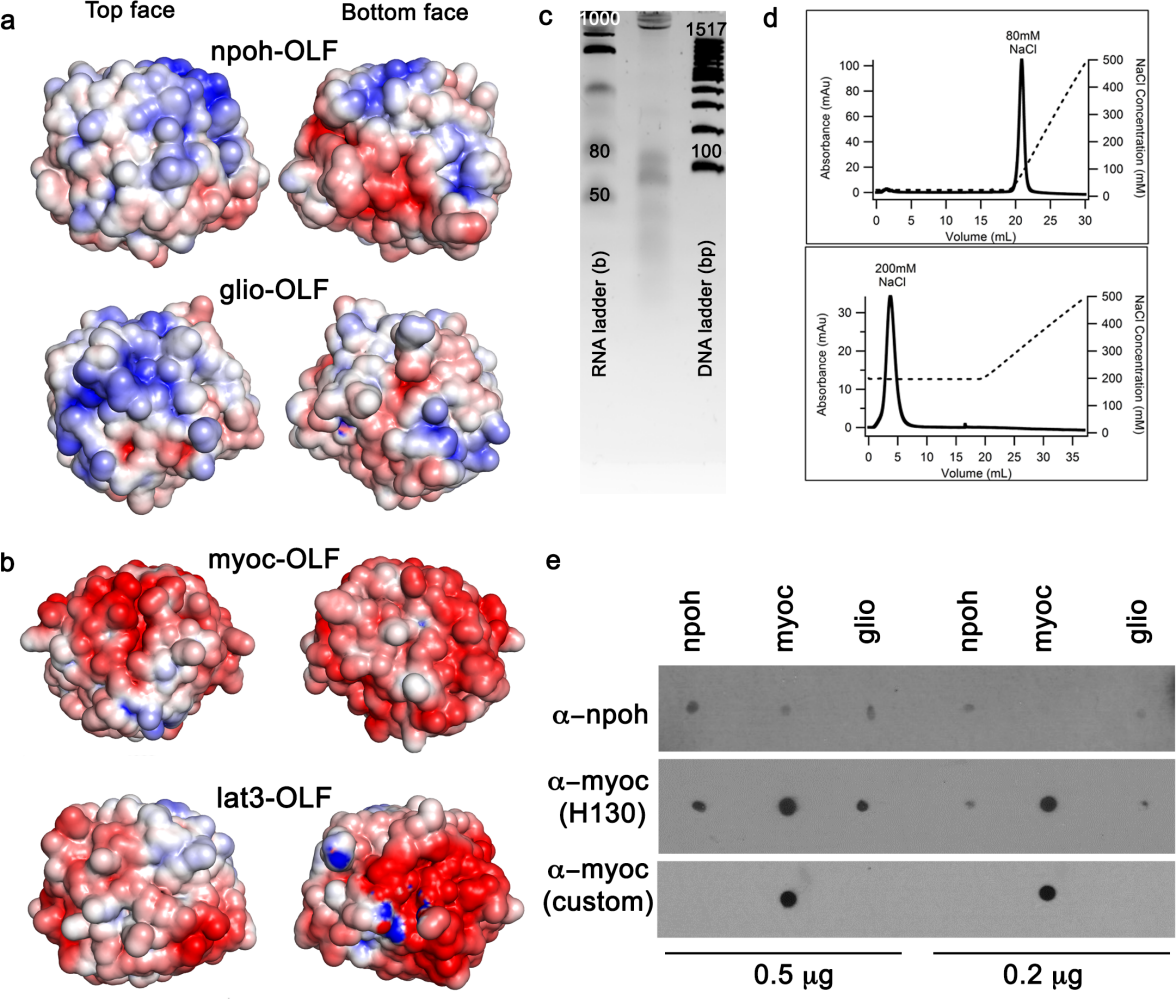
Electrostatic surface representations and biochemical analysis of nucleotide and heparin binding for npoh-OLF. (a) Electrostatic surfaces of npoh-OLF and glio-OLF at top and bottom faces. (b) Electrostatic surfaces of myoc-OLF (PDB code 4WXU) and lat3-OLF (PDB code 5AFB). Surface potentials are colored negative (red, -5 kT/e^-^) to positive (blue, + 5 kT/e^-^). (c) Extraction analysis reveals small nucleotide stretches bound to npoh-OLF. (d) Low affinity binding of npoh-OLF to heparin column; no binding occurs with buffers at physiological ionic strength. (e) Commercial antibodies, anti-npoh-OLF and anti-myocilin (H130), lack specificity and detect npoh-OLF, myoc-OLF, and glio-OLF compared to custom myocilin antibody prepared in this study.

Inspection of the electrostatic surface potentials (Fig 4A and 4B) reveals striking differences among glio-OLF, npoh-OLF, myoc-OLF and lat3-OLF (calculated pIs for structural domains are ~8.1, 7.3, 5.0, and 5.4 respectively). In contrast to the negative surface potential of myoc-OLF [12] and lat3-OLF (Fig 4B), those of glio-OLF and npoh-OLF are predominately positive on the top surface while both negative and positive on the bottom surface (Fig 4A). For npoh-OLF, 18 of 20 total Arg and Lys residues are solvent exposed, with a large positive region concentrated at the top face of blades A and B and extending into two small positive patches involving Arg 400 of the short helical turn and Lys 423 of the loop between strands D-16 and D-17. The bottom surface of npoh-OLF consists of two small positive areas, one near the disulfide bond and another involving strand B-10, with a substantial negative patch composed of Asp/Glu rich loops between strands A-3/A-4 and strands E-19/E-20, where one of two fortuitously bound glycerol molecules is modeled in the npoh-OLF structure (not shown). For glio-OLF, all 24 Arg and Lys residues are exposed to solvent with a large positive patch on the top face of blades B, C, and D, but arranged distinctly from npoh-OLF (Fig 4A). The bottom surface of glio-OLF consists of a small positive region involving the N-terminus and Arg 476 of the cation-π interaction near the molecular clasp (see above), while a large negative area involving Asp/Glu residues of blade C extends into blade E and includes the loop connecting strands C-11 and C-12 (Fig 4A). In this region, a glycerol is modeled in chain B of our glio-OLF structure (not shown), close to the position of a modeled methyl-2-pentane-diol molecule in rat glio-OLF [17].

We hypothesized that the npoh-OLF positive surface charge could indicate affinity for negatively charged nucleotides, glycosaminoglycans (GAGs), or lipids. Structure based computational programs predict both heparin [55] and DNA [56] binding (not shown). Experimentally, recombinant npoh-OLF and glio-OLF copurify with nucleic acids unless stringently removed, and biochemical extraction of nucleotides bound to npoh-OLF reproducibly yields a range of small polynucleotides (Fig 4C). Heparin binding, however, is very weak. Increases in npoh-OLF thermal stability in the presence of GAGs are modest (Table 2), and while npoh-OLF devoid of bound nucleotides can bind a heparin column, this is only in buffers with non-physiological, low ionic strength (Fig 4D). We note that by comparison, myoc-OLF does not bind the heparin column under any experimental conditions tested (not shown). Lastly, npoh-OLF does not appear to have affinity for membrane lipids (not shown). It is tempting to speculate that nucleotide binding plays a biological role in neuronal OLFs like npoh-OLF and glio-OLF. Such binding is unprecedented in the literature, but could be biological, given that our analysis indicates it is not an obvious proxy for GAG or lipid binding. For example, nucleotides could bind to the positively charged patches found at the top or bottom face of npoh-OLF and/or glio-OLF. Such roles could involve purinergic signaling [57], or perhaps chaperoning of extracellular microRNAs, which are also emerging players in neuronal development [58, 59].

Taken together, our experimental findings that OLF structures exhibit divergent surface residues, loop structures, and electrostatic potentials favor the implication that subfamily members likely have distinct binding partners, and thus non-redundant function. A contributing factor to the historical difficulty in assigning specific biological roles to OLFs is the fact that commercially available antibodies to study OLF domain containing proteins are not very selective. Indeed, in our hands, two such antibodies, one for noelin/olfactomedin-1 and another for myocilin (H130), which are targeted to long peptide sequences in their respective N- or C-terminal regions where there is significant sequence similarity among OLF domains (S1 Fig), recognize npoh-OLF, myoc-OLF, and glio-OLF (Fig 4E). We posited that it should be possible to generate more selective reagents through structure-based design. In our first such example, to assist in ongoing myocilin mouse studies, we raised an antibody to a surface exposed, largely non-conserved, short soluble peptide stretch, R_332_YELDTETVKAEKEIPGA. This *M*. *musculus* myocilin amino acid sequence differs from *H*. *sapiens* only at one position (Asp 336 is Glu 350 in myocilin). Indeed, this yielded a new polyclonal antibody selective for myoc-OLF over the other two purified OLF proteins available in our lab (Fig 4E). Although additional experiments are needed to test the selectivity of this antibody in cell lines and animal models, the overall approach could be applied similarly to generate selective antibodies against other OLF domains. In sum, the availability of phylogenetically distinct OLF structures expands our appreciation of the physicochemical diversity of the OLF domain, paving the way for better methods to interrogate reported protein-protein interactions, discover new binding partners, and investigate molecular functions of OLF domain-containing proteins.

## Supporting Information

S1 Fig Multiple sequence alignment of npoh-OLF, glio-OLF, myoc-OLF, and lat3-OLF. Arrows: β-strands; T: turns; spiral feature, α-helices. White residues with black background: identical residues; boxed residues colored black, similar; asterisk, cysteine residue present in npoh-OLF, myoc-OLF, and lat3-OLF but absent in glio-OLF; underline: myocilin peptide stretch for custom antibody.
(PDF)

S1 Table Primers used in this study. (PDF)
