# Supplementary material for: Molecular Details of Olfactomedin Domains Provide Pathway to Structure-Function Studies
Source: PLoS One. 2015 Jun 29;10(6):e0130888. doi: 10.1371/journal.pone.0130888 (PMC4488277; doi:10.1371/journal.pone.0130888)
Supplement: S1 Fig — Arrows: β-strands; T: turns; spiral feature, α-helices. White residues with black background: identical residues; boxed residues colored black, similar; asterisk, cysteine residue present in npoh-OLF, myoc-OLF, and lat3-OLF but absent in glio-OLF; underline: myocilin peptide stretch for custom antibody. (PDF) [file pone.0130888.s001.pdf]

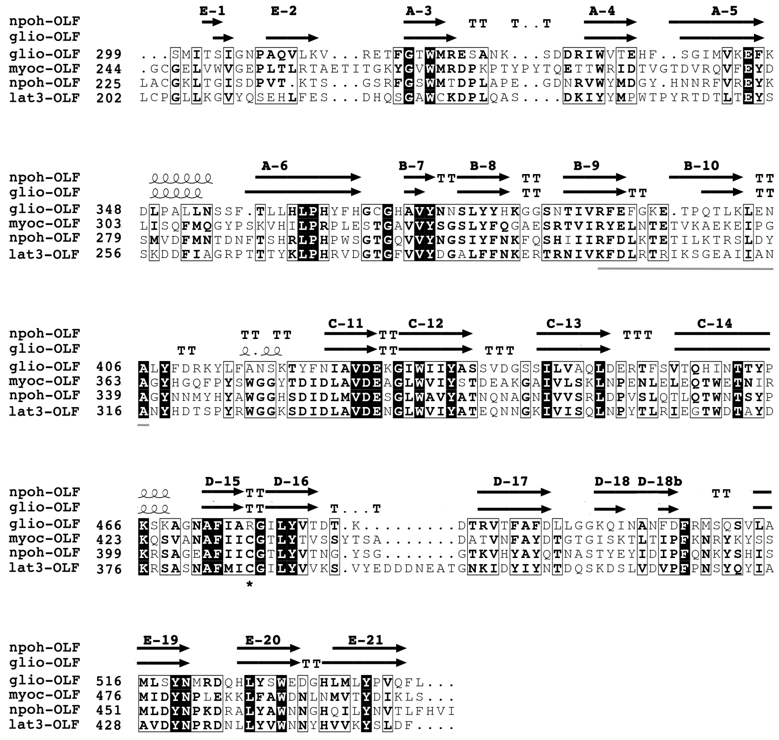

S1 Fig. Multiple sequence alignment of npoh-OLF, glio-OLF, myoc-OLF, and lat3-OLF. Arrows: β-strands; T: turns; spiral feature, α-helices. White residues with black background: identical residues; boxed residues colored black, similar; asterisk, cysteine residue present in npoh-OLF, myoc-OLF, and lat3-OLF but absent in glio-OLF; underline: myocilin peptide stretch for custom antibody.
