# Supplementary material for: Molecular Details of Olfactomedin Domains Provide Pathway to Structure-Function Studies
Source: PLoS One. 2015 Jun 29;10(6):e0130888. doi: 10.1371/journal.pone.0130888 (PMC4488277; doi:10.1371/journal.pone.0130888)
Supplement: S1 Table — (PDF) [file pone.0130888.s002.pdf]

S1 Table: Primers used in this study.

**<u>Npoh-OLF Cloning</u>**			
Plasmid Template	Target Vector		Primer Used
pcDNA (Open Biosystems)	pET-30 Xa/LIC	Forward	5'-GGTATTGAGGGTCGCCTCCGTGCATGC
		Reverse	5'-AGAGGAGAGTTAGAGCCTCATCACAACTCGTC
pET-30 Xa/LIC	pMAL-c4x	Forward	5'-CGCCGAGCTCTATTGAGGGTCGC
		Reverse	5'-GCCGAATTCAGGAGAGTTAGAGCCTTATCA
Npoh-OLF (C221G)	Mutagenesis	Forward	5' GGTCGCCTCCGTGCAGGCATGCAAAAACTAGC
**<u>Glio-OLF Cloning</u>**			
Plasmid Template	Target Vector		Primer Used
pcDNA (Open Biosystems)	pET-30 Xa/LIC	Forward	5'-GGTATTGAGGGTCGCGATACCTTGGTG
		Reverse	5'-AGAGGAGAGTTAGAGCCTCATCACCGCTG
pET-30 Xa/LIC	pMAL-c4x	Forward	5'-CGCCGAGCTCTATTGAGGGTCGC
		Reverse	5'-GCCGAATTCAGGAGAGTTAGAGCCTTATCA
